# The Influence of Daily Honey-Sweetened Yogurt Intake on Outcomes of Low-Grade Inflammation and Microbial Metabolites in Postmenopausal Women

**DOI:** 10.3390/nu18030522

**Published:** 2026-02-04

**Authors:** Yuyi Chen, Valentina Medici, Carl L. Keen, Roberta R. Holt

**Affiliations:** 1 Department of Nutrition, University of California Davis, Davis, CA 95616, USA; uyichen@ucdavis.edu (Y.C.); clkeen@ucdavis.edu (C.L.K.); 2 Division of Gastroenterology and Hepatology, Department of Internal Medicine, University of California Davis, Sacramento, CA 95817, USA; vmedici@health.ucdavis.edu

**Keywords:** honey, cytokines, short chain fatty acids, microbiome, cardiometabolic

## Abstract

**Background/Objectives**: After fermentation, yogurt is often supplemented with probiotics, yet sweetened with added sugars that can negatively impact cardiometabolic health. Honey provides rare sugars, oligosaccharides and phenolics that may promote gut and cardiometabolic health. We aimed to determine the impact of yogurt sweetened with commercial clover blossom honey on pro-inflammatory Th17 cytokines and microbial-derived metabolites in healthy postmenopausal women. **Methods**: In a randomized controlled crossover dietary intervention trial, postmenopausal women (45–65 years of age) consumed two 150 g servings of yogurt for breakfast for 4 weeks, with each serving sweetened with a tablespoon of clover blossom honey or an isocaloric amount of sugar. Blood samples were collected for the measurement of plasma lipids, bile acids (BA) and Th17 cytokines, along with fecal short-chain fatty acids (SCFA). The primary outcome was plasma interleukin (IL)-23. **Results**: Neither dietary intervention significantly changed IL-23, plasma lipids, fecal SCFA or plasma BA. Compared to sugar-sweetened yogurt, IL-33 was significantly lower after 4 weeks of honey-sweetened yogurt intake. **Conclusions**: In a healthy population of postmenopausal women, the daily intake for 4 weeks of honey-sweetened yogurt did not significantly impact our primary outcome of IL-23. Instead, lower plasma levels of IL-33 were observed with honey compared to sugar-sweetened yogurt intake. The impact of the intervention on this cytokine was independent of changes in fecal SCFA and plasma BA. Confirmatory studies, in a larger population with levels of honey intake within dietary recommendations for added sugar, are warranted.

## 1. Introduction

The inclusion of yogurt within a healthy dietary pattern contributes to cardiometabolic health [1,2], possibly through impacts on blood pressure and chronic inflammation [2,3]. Metabolic diseases are often characterized by a low-grade systemic inflammation originating from the adipose tissue in a maladaptive response to physiological stress and overnutrition [4], with the intestinal microbiome contributing to this response [5,6]. As both an endocrine and immune organ, a healthy gut microbiome produces an array of metabolites that maintain the gut epithelial barrier, promote immune tolerance, and influence systemic homeostasis [5,7,8]. Gut dysbiosis profoundly changes the production of metabolites, such as short-chain fatty acids (SCFA) that can alter gut barrier integrity and allow gut derived pro-inflammatory mediators to enter the circulation. Cardiometabolic risk factors, such as obesity, dyslipidemia and blood pressure, have all been associated with gut dysbiosis along with increased metabolic endotoxemia and gut permeability [7,9,10]. Healthy dietary patterns promote gut microbiome health, while calorically dense, nutrient-poor diets that are high in sodium promote dysbiosis. In both animal models and human subjects, a high salt diet induces hypertension by reducing levels of beneficial intestinal bacterial populations, while increasing the abundance of tissue and circulating pro-inflammatory CD4^+^ T helper (Th)17 cells, with interleukin (IL)-23 as a key regulator of this response [7,11]. Pathogenic differentiation of Th17 cells by IL-23, along with IL-6 and transforming growth factor beta, drives IL-17 secretion that promotes vascular dysfunction and hypertension [12].

After fermentation, yogurt is often supplemented with probiotics yet sweetened with added sugars that can negatively impact cardiometabolic health [13]. Glucose and fructose are the major sugars found in the natural sweetener honey. When added to yogurt, honey improves probiotic survivability in vitro, and probiotic enrichment after daily yogurt intake [14,15]. These observations may be the result of the provision of rare sugars, oligosaccharides and phenolics from honey [16,17]. A meta-analysis of 18 controlled trials reports of positive impacts of honey intake on glucose control and plasma lipids [18], yet the role of the gut microbiome in this response is currently unknown. In this study, we aim to determine the impact of yogurt sweetened with commercial clover blossom honey on pro-inflammatory Th17 cytokines and microbial-derived metabolites in healthy postmenopausal women. Immune system changes occurring during menopause include increased Th17 cytokine levels that promote chronic inflammatory disease such as cardiovascular disease (CVD) and osteoporosis [19]. We suggest that honey-sweetened yogurt will lower pro-inflammatory cytokines, specifically our primary outcome IL-23, through changes in the gut microbiome that increase SCFA and secondary bile acids (BA).

## 2. Materials and Methods

### 2.1. Participants

Healthy women, 45–65 years of age, and having a BMI of 25–30 kg/m^2^ were recruited from the greater Sacramento, California Metropolitan Area, USA. All study participants were postmenopausal for a minimum of two years. Volunteers were excluded for the presence of cardiovascular, diabetes, metabolic, liver, and kidney disease. Cancer within the previous 5 years, malabsorption, food allergies, a history of serious illness, use of prescription medications, or currently under acute medical care were also exclusionary. Lifestyle factors resulting in exclusion included the current use of supplements or an unwillingness to stop at least 1 month prior to study entrance. The regular intake of a high “plant”-based diet was also exclusionary, to include the daily consumption of a vegetarian or vegan-type diets, concentrated food supplements and extracts, and fruit intake (including juices) greater than 2 cups a day, and vegetable intake greater than 3 cups per day. This intervention was conducted between September 2022 and July 2025 and was registered at ClinicalTrials.gov (NCT04248127), with the protocol approved by the Institutional Review Board of the University of California (UC), Davis. All participants gave their informed consent before they participated in the clinical screening.

### 2.2. Study Design

Eligible participants were randomized by block design and enrolled into a single blind, two arm dietary intervention trial in a crossover design. Each intervention arm was 4 weeks in length, with a 4 week washout period between the two arms. The participants were randomized to consume either two morning servings (serving = 150 g) of yogurt sweetened with 1 tablespoon (tbsp) per serving of clover blossom honey, or a yogurt sweetened with an isocaloric amount of cane sugar every day. After 4 weeks of no yogurt intake, the participants were provided with the yogurt they had not been assigned in the first arm. Participants were instructed to consume yogurt in replacement of or as part of breakfast, or as a late morning snack. They were also instructed to not add anything to the yogurt or consume any additional yogurt or honey throughout the 12 week study period. For compliance, the participants were asked to fill out a daily intake log and return this log each week when they picked up another week supply of yogurt.

Study outcomes were measured before and after each study arm. Twenty-four to 48 h prior to each study visit, the study participants were asked to collect a stool sample at home and to keep it frozen until their scheduled study visit. On the morning of each study visit, the study participants arrived at the facility after an overnight fast. Anthropometric measures were collected. After a 15 min rest in a seated position, three blood pressure measurements each five minutes apart were collected using an automated oscillometric unit (Vital Spot, VSM 300, Welch Allyn, Skaneateles Falls, NY, USA). Following the blood pressure measurements, a blood sample was collected by the study nurse.

### 2.3. Test Foods

The base yogurt was Chobani’s plain Greek non-fat yogurt fermented by *Streptococcus thermophilus*, *Lactobacillus bulgaricus*, and supplemented with *Lactobacillus acidophilus*, *Bifidobacterium lactis*, *Lactobacillus casei*, and *Lactobacillus rhamnosus*. The honey was commercially available US Grade A Clover Blossoms Honey 2021 with a use-by date of 11/22/2024 (Dutch Gold Honey, Inc., Lancaster, PA, USA), and provided by the National Honey Board (Erie, CO, USA). The honey was received in January 2022, portioned into airtight and dark containers, and frozen at −20 °C within 48 h of receipt. For each one-week supply of yogurt, enough honey was thawed in a refrigerator overnight or at room temperature the morning of mixing. One tbsp of honey (21 g) was used per 150 g serving of yogurt, with a tbsp of honey providing 17 g of added sugar. A comprehensive characterization of the honey has been previously described [16], with major phenolics and sugar composition of the honey for a 2 tbsp daily serving presented in Table 1. The control yogurt was sweetened with an isocaloric amount of cane sugar. A daily serving of yogurt provided 279 kcal, 28 g of protein, 344 mg of calcium and 115 mg of sodium.

### 2.4. Study Outcomes

Clinical outcomes measures included a complete blood count, a comprehensive metabolic panel, and a lipid panel analyzed by UC Davis School of Medicine’s Department of Pathology and Laboratory Medicine (Sacramento, CA, USA). Uric acid was measured using a colorimetric assay according to the manufacturer’s instructions (Abcam, Boston, MA, USA).

Plasma Th17 cytokines were analyzed with a Luminex Milliplex Human Th17 premixed 25 plex magnetic bead panel (Millipore Sigma, St. Louis, MO, USA) by the UC Davis Intellectual and Developmental Disabilities Research Center’s Biological and Molecular Analysis Core. Luminex methodology with bead immunoassays was employed to measure circulating plasma levels of IL-1 beta, -4, -6, -10, -17a, -17f, -21, -22, -23, -31, -33, interferon (IFN)-γ, and tumor necrosis factor (TNF)-alpha. Briefly, analyte-specific antibody conjugated beads were incubated with a 25 µL assay buffer and 25 μL of plasma on an orbital shaker in the dark. Following incubation, the fluid was gently aspirated by vacuum manifold and the beads washed. Detection antibodies (25 μL) were then added to each well and incubated on an orbital shaker for 1 h at room temperature. Subsequently, 25 μL of Streptavidin-Phycoerythrin was added to each well and incubated on a plate shaker for 30 min at room temperature. The plate was then washed 3 times, 150 μL of 1× sheath fluid was added to each well, and the fluorescence was measured on a Bio-Plex 200 System (Bio-Rad Laboratories Inc., Hercules, CA, USA).

Fecal SCFA and plasma primary and secondary bile acids (BA) were measured by LC-MS/MS in accordance with Metabolon’s validated methodologies (Metabolon, Morrisville, NC, USA). Fecal samples were spiked with stable labeled internal standards, homogenized, and subjected to protein precipitation with an organic solvent. After centrifugation, an aliquot of the supernatant was derivatized and injected into an Agilent 1290/SCIEX (Agilent Technologies, Santa Clara, CA, USA) QTRAP 5500/6500+ LC-MS/MS system (SCIEX, Framingham, MA, USA) equipped with a C18 reversed phase UHPLC column. Mass spectrometry was operated in negative mode using electrospray ionization (ESI). The peak area of the individual analyte product ions was measured against the peak area of the product ions of the corresponding internal standards. Quantitation was performed using a weighted linear least squares regression analysis generated from fortified calibration standards prepared concurrently with study samples.

For plasma BA analysis, calibration samples were prepared at eight different concentration levels and spiked into a phosphate-buffered saline/bovine serum albumin (PBS/BSA) solution with corresponding calibration spiking solutions. Calibration samples, study samples, and quality control samples were spiked with a solution of isotopically labeled internal standards and subjected to protein precipitation with an organic solvent (acidified methanol). Following centrifugation, an aliquot of the organic supernatant was evaporated to dryness in a gentle stream of nitrogen. The dried extracts were reconstituted and injected onto an Agilent 1290 Infinity I or II/SCIEX 6500 or 6500+ Triple Quadrupole or QTRAP LC-MS/MS system equipped with a C18 reverse phase UHPLC column. Mass spectrometry was operated in negative mode using electrospray ionization (ESI). The peak area of each bile acid parent (pseudo-MRM mode) or product ion was measured against the peak area of the respective internal standard parent (pseudo-MRM mode) or product ion. Quantitation was performed using a weighted linear least squares regression analysis generated from fortified calibration standards prepared concurrently with study samples. LC-MS/MS raw data was collected using SCIEX software Analyst 1.7.3 and processed using SCIEX OS-MQ software v3.1.6.

### 2.5. Statistical Analysis

#### 2.5.1. Sample Size Calculation

As a key regulator of Th17 response, IL-23 [11] was used as the primary outcome. In an a priori power analysis using a mean difference of 28.9 pg/mL and an SD of 30 pg/mL, we determined that 18 participants were needed to complete the trial to achieve 80% power and α = 0.05.

#### 2.5.2. Data Preparation

Cytokine, SCFA, and BA concentrations were assessed for skewness. Variables with |skewness| > 1.0 were log-transformed using log(x + 1). Ultra-sparse cytokines (>70% zeros: IL-17E/IL-25, IL-31, IL-17F) were converted to binary detection variables (detected vs. not detected).

Missing values were equal to or less than 5%. Multiple imputation by chained equations (MICE) was performed using the mice package [20] with 15 imputed datasets (m = 15) and 20 iterations. Predictive mean matching was used with a predictor matrix enhanced for the crossover design structure (minimum correlation = 0.3). Results were averaged across imputed datasets, with binary variables rounded to 0 or 1 and log-transformed variables back-transformed to original scales.

Elevated IL-6 levels in menopausal women have been associated with CVD risk and severity [21,22,23]. As an exploratory aim, participants were further classified the study participants into high vs. low IL-6 groups based on baseline (visit 1 of first period) median IL-6 levels.

#### 2.5.3. Statistical Tests and Models

Outcomes were stratified by baseline median IL-6 levels for descriptive analyses. We used Fisher’s exact to examine differences in categorical variables due to small sample size, independent *t*-test for normally distributed continuous variables, and Mann–Whitney U test for non-normally distributed continuous variables.

For primary analysis, we used linear mixed-effects models with restricted maximum likelihood estimation:

$$\begin{array}{c} Y_{ij}=\beta_{0}+\beta_{1}{\mathrm{Intervention}}_{i}+\beta_{2}{\mathrm{Time}}_{j}+\beta_{3}{\left(\mathrm{Intervention}\times \mathrm{Time}\right)}_{ij}+\beta_{4}{\mathrm{Sequence}}_{i}\\ +\beta_{5}{\mathrm{Period}}_{j}+\beta_{6}{\mathrm{Weight}}_{ij}+u_{i}+\epsilon_{ij} \end{array}$$

where $Y_{ij}$ is the outcome for subject i at time j, Intervention is intervention group (honey- vs. sugar-sweetened yogurt), Time is study visit (visit 1, visit 2), Sequence is intervention order (AB vs. BA), Period controls for time effects, Weight is a covariate (excluded for BMI models), $u_{i}$ is the random subject intercept, and $\epsilon_{ij}$ is residual error. The intervention × time interaction ($\beta_{3}$) was the primary parameter of interest, representing differential change between interventions. For binary outcomes (cytokine detection), we used generalized linear mixed-effects models with binomial family and logit link. Identical models were applied to participants with high inflammatory burden (*n* = 10) as a sensitivity analysis.

We performed Principal Component Analysis (PCA) on significant outcomes from mixed-effects models (SCFAs: acetic, propionic, butyric, valeric, hexanoic acids; BA: glycocholic, taurocholic, taurochenodeoxycholic, tauroursodeoxycholic acids; clinical outcomes: SBP and DBP). Non-normal variables (|skewness| > 1.0) were transformed using log(x + 1) or Johnson transformations, selecting the method yielding lower absolute skewness. Variables were standardized (mean = 0, SD = 1) before analysis. Biplots display 95% confidence ellipses for group comparisons. Permutational Multivariate Analysis of Variance (PERMANOVA) was performed on Euclidean distance matrices using 9999 permutations to test for intervention and IL-6 group effects on multivariate metabolic profiles. Effect sizes (R^2^) and *p*-values are reported, with R^2^ > 0.01, >0.05, and >0.10 indicating small, medium, and large effects.

All analyses were conducted in R (v4.2.1). Statistical significance was set at α = 0.05 (two-tailed). *p*-values for fixed effects used Satterthwaite’s approximation for degrees of freedom. Results are reported as β coefficients with 95% confidence intervals (CI = β ± 1.96 × SE).

## 3. Results

### 3.1. Baseline Characteristics and Dietary Analysis

**Figure 1:**
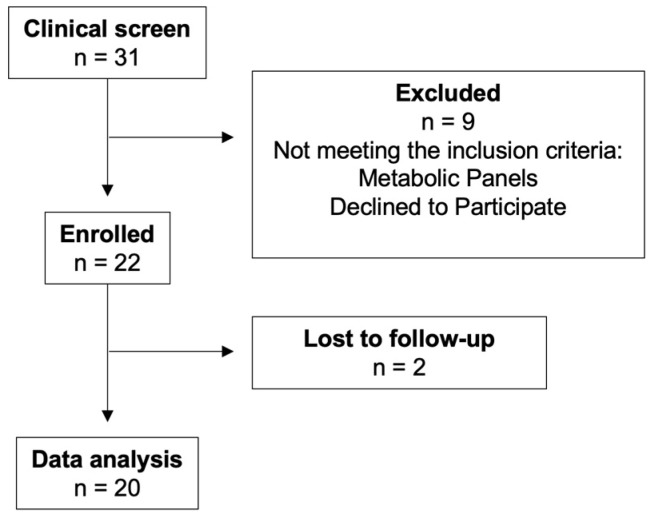
CONSORT Flow Diagram of Participant Recruitment and Retention.

A total of 20 participants (high IL-6, *n* = 10; low inflammatory burden, *n* = 10) completed the crossover trial and were included in the analysis (Figure 1). The study participants had a mean age of 60.1 ± 4.9 years and mean BMI of 28.4 ± 2.74 kg/m^2^ (Table 2). Mean baseline SBP was 114.4 ± 8.6 mmHg and DBP was 74.7 ± 8.5 mmHg. Although blood pressure was within a normal range, the study population had borderline high total- and LDL- cholesterol levels, with fasting blood glucose levels in the upper range of normal. Baseline clinical outcomes between those grouped into either low or high IL-6 were not significantly different (*p* > 0.05).

Baseline cytokine profiles demonstrated significant differences within the study population (Supplementary Table S1). Participants with higher than the median in IL-6 (IL-6 > 4.41 pg/mL) had significantly higher levels for multiple cytokines compared to those with lower IL-6 levels. Notably, higher levels of IL-10, IL-13, IL-4, IL-5, TNF-β, and IL-28A were observed, in many cases more than 10-fold (all *p* < 0.05). IL-23 was also significantly elevated, though to a lesser degree (*p* = 0.037). Additionally, IL-31 was detected exclusively in participants in the high IL-6 group (60.0% vs. 0%, *p* = 0.011).

Compliance with the yogurt intervention was greater than 95%. No significant differences in any macronutrient or micronutrient intake were observed between intervention periods (all *p* > 0.05) (Table 3). Minus the yogurt products, mean daily energy intake was 1628.5 ± 427.4 kcal during the honey-sweetened yogurt period and 1600.0 ± 357.2 kcal during the sugar-sweetened yogurt period. Macronutrient distribution was comparable between periods: protein (64.9 vs. 67.7 g/day), carbohydrate (172.1 vs. 159.9 g/day), added sugar (29.7 vs. 24.9 g/day), total fiber (16.1 vs. 14.2 g/day), and total fat (71.3 vs. 70.8 g/day) for honey and sugar periods, respectively.

### 3.2. Clinical Outcomes

**Figure 2:**
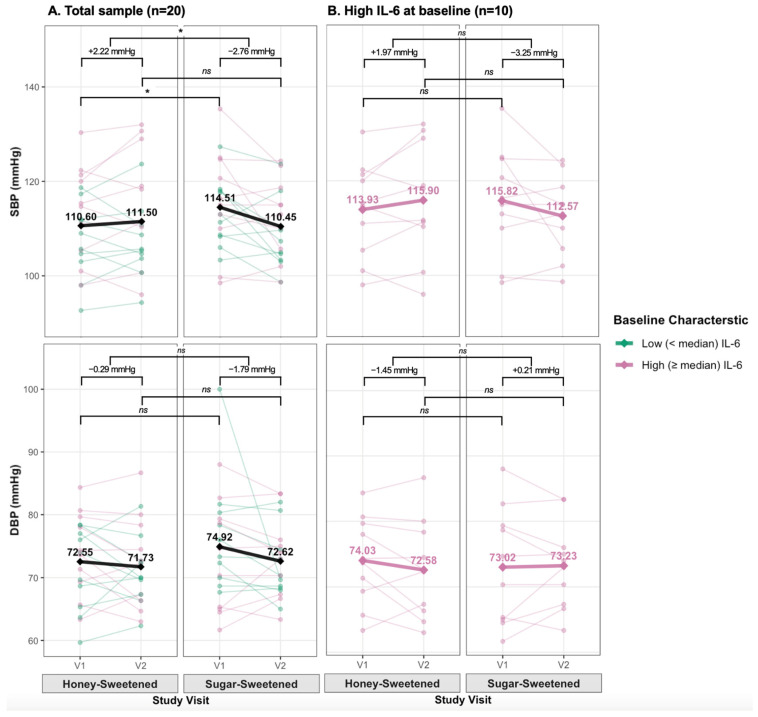
Subject-level changes in blood pressure by intervention and baseline IL-6 levels. Individual participant trajectories for systolic (SBP) and diastolic blood pressure (DBP) across visits in (**A**) total sample (*n* = 20) and (**B**) high IL-6 subgroup (*n* = 10). Green/Pink lines represent individual trends for those classified into IL-6 subgroups of “low” < or “high” ≥ median IL-6 at baseline. Bold lines show period means with adjusted mean changes labeled. Results from linear mixed-effects models adjusted for time, sequence, period, and weight, with honey period as reference. Significant intervention-by-time interaction for SBP (*p* = 0.029) in total sample; no significant DBP effects. ns = not significant. * *p* < 0.05.

Table 4 presents descriptive statistics and mixed-effects model results examining the effects of honey-sweetened versus sugar-sweetened yogurt on clinical outcomes. At baseline, those assigned the sugar-sweetened yogurt had a higher SBP than when they were assigned honey-sweetened yogurt (adjusted difference for intervention = 3.44 mmHg, *p* = 0.035). A significant intervention-by-time interaction for lower SBP with sugar-sweetened yogurt intake was observed (adjusted differential change in SBP = −4.98 mmHg (95% CI: −9.34 to −0.62, *p* = 0.029). This included non-significant trends over time for a 2.22 mmHg increase in SBP with honey-sweetened yogurt intake and a 2.76 mmHg reduction in SBP after sugar-sweetened yogurt intake (adjusted difference for time = 2.22 mmHg, *p* = 0.189; Table 4 and Figure 2A, left panel). Sequence (intervention order) effect was not significant (*p* = 0.273). However, a significant period effect was also detected (adjusted difference = −1.28 mmHg per period, *p* = 0.028), with lower values in the second period compared to the first period regardless of intervention assignment (Supplementary Figure S1A). In contrast, no significant main or interactive effects were observed for DBP (Figure 2A). Additionally, no significant effects for both SBP and DBP were observed in participants stratified by IL-6 level (*p* > 0.05) (Figure 2B). Plasma HDL cholesterol levels significantly decreased over time from visit 1 to visit 2 (adjusted difference = −4.45 mg/dL, *p* = 0.016). No significant intervention or interaction effects were observed for all lipid measures (all *p* > 0.05; Table 4).

### 3.3. Cytokines

**Figure 3:**
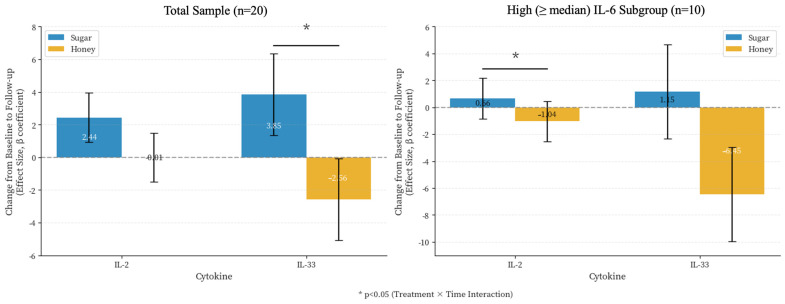
Differential effects of honey- versus sugar-sweetened yogurt on inflammatory cytokines. Adjusted mean changes (β coefficients) in IL-2 and IL-33 across visits in total sample (*n* = 20) and high IL-6 subgroup (high IL-6 group (“low” < or “high” ≥ median IL-6) at baseline; *n* = 10). Blue/orange bars indicate sugar/honey-sweetened yogurt; error bars show standard errors. Results from linear mixed-effects models adjusted for time, sequence, period, and weight, with honey period as reference. Significant intervention-by-time interactions for IL-33 (total sample, *p* = 0.044) and IL-2 (high inflammatory burden, *p* = 0.028). * *p* < 0.05.

No significant main effects in IL-23 levels were observed, with an intervention-by-time interaction approaching significance (adjusted differential change = 0.346 pg/mL, *p* = 0.056). Most of the remaining cytokines also did not significantly change with either intervention except for IL-33. A significant intervention-by-time interaction was observed for IL-33 (adjusted difference = 6.42 pg/mL (95% CI: 0.18 to 12.66), *p* = 0.044), with plasma levels increasing after sugar-sweetened yogurt intake (+3.85 pg/mL) and decreasing with honey-sweetened yogurt intake (−2.56 pg/mL) (Figure 3, left panel). No significant interaction was observed for IL-2 in the total sample (adjusted difference = 2.45 pg/mL, *p* = 0.119). However, a significant interactive effect for IL-2 (adjusted difference = 1.70 pg/mL (95% CI: 0.19 to 3.21), *p* = 0.028) was observed for those in the high IL-6 group. IL-2 levels increased (+0.66 pg/mL units) after the intake of sugar-sweetened yogurt by these individuals, while IL-2 levels were lower (−1.04 units pg/mL) after 4 weeks of honey-sweetened yogurt intake (Figure 3, right panel). The IL-33 interaction in this subgroup did not reach significance (adjusted difference = 7.60 mg/dL, *p* = 0.119), though directional patterns were consistent with the total sample.

### 3.4. Fecal Short-Chain Fatty Acids

Baseline (visit 1) fecal SCFA levels were significantly higher for the sugar-sweetened compared to the honey-sweetened yogurt arm for acetic acid (adjusted difference = −654 µg/g, *p* = 0.015), butyric acid (adjusted difference = −571 µg/g, *p* = 0.013), propionic acid (adjusted difference = −430 µg/g, *p* = 0.010), valeric acid (adjusted difference = −107 µg/g, *p* = 0.008), and hexanoic acid (adjusted difference = −62 µg/g, *p* = 0.005) (Supplementary Figure S2A). Differences in SCFA levels were generally larger amongst participants in the high IL-6 group (average +41 µg/g), though only acetic acid (adjusted difference = −758 µg/g, *p* = 0.044) and hexanoic acid (adjusted difference = −85 µg/g, *p* = 0.016) reached statistical significance.

**Figure 4:**
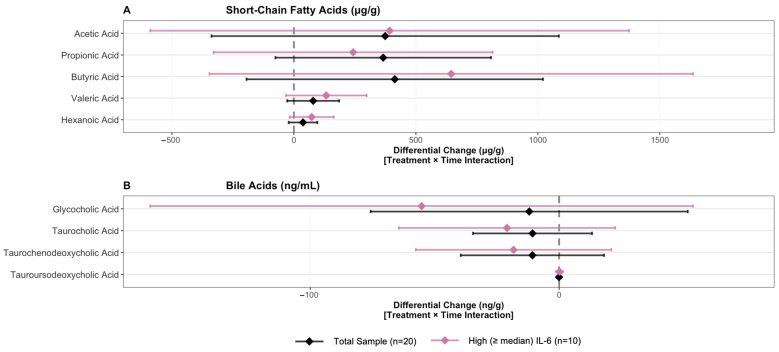
Differential Changes in Fecal Short-Chain Fatty Acids and Bile Acids Between Honey- and Sugar-Sweetened Yogurt Intervention. Intervention-by-time interaction effects with 95% confidence intervals comparing honey- vs. sugar-sweetened yogurt effects on (**A**) fecal short-chain fatty acids (µg/g) and (**B**) bile acids (ng/mL). Black: total sample (*n* = 20); Pink: IL-6 subgroup (“low” < or “high” ≥ median IL-6 at baseline; *n* = 10). Dashed line indicates no effect (β = 0). Results from linear mixed-effects models adjusted for time, sequence, period, and weight, with honey period as reference. Positive β indicates greater increase (or smaller decrease) in sugar vs. honey period. Error bars represent 95% confidence intervals. All interactions non-significant (*p* > 0.05).

For all participants, fecal SCFA levels decreased from visit 1 to visit 2 but were not significantly different by visit 2 (all *p* > 0.05). An opposing temporal pattern was observed between the two interventions with fecal SCFA concentrations decreasing with honey-sweetened yogurt intake (acetic acid: −327 µg/g, *p* = 0.236; butyric acid: −342 µg/g, *p* = 0.148; propionic acid: −253 µg/g, *p* = 0.142; valeric acid: −44 µg/g, *p* = 0.290; hexanoic acid: −10 µg/g, *p* = 0.663) while increasing with sugar-sweetened yogurt intake (acetic acid: +47 µg/g; butyric acid: +72 µg/g; propionic acid: +113 µg/g; valeric acid: +35 µg/g; hexanoic acid: +27 µg/g). Intervention-by-time interactions were positive but not significant (acetic acid: adjusted differential change = 374 µg/g, *p* = 0.308; butyric acid: 413 µg/g, *p* = 0.188; propionic acid: 366 µg/g, *p* = 0.110; valeric acid: 79 µg/g, *p* = 0.152; hexanoic acid: = 37 µg/g, *p* = 0.219), showing a consistent pattern of differential changes between interventions (Figure 4A). This convergence pattern was consistent in the high IL-6 subgroup (acetic acid: adjusted differential change = 392 µg/g, *p* = 0.440; butyric acid: 645 µg/g, *p* = 0.214; propionic acid: 243 µg/g, *p* = 0.413; valeric acid: 133 µg/g, *p* = 0.129; hexanoic acid: = 73 µg/g, *p* = 0.125), though none reached statistical significance.

A significant period effect was observed for hexanoic acid (adjusted difference = −16.4 µg/g, *p* = 0.036), with lower concentrations in the second period compared to the first period, regardless of intervention (Supplementary Figure S1B). This pattern was similar to that observed for SBP (Supplementary Figure S1A).

### 3.5. Bile Acids

Plasma BA concentrations were significantly higher at baseline (visit 1) for the sugar-sweetened compared to the honey-sweetened yogurt arm: glycocholic acid (adjusted difference = 47.48 ng/mL, *p* = 0.046), taurocholic acid (19.80 ng/mL, *p* = 0.027), taurochenodeoxycholic acid (18.69 ng/mL, *p* = 0.081), and tauroursodeoxycholic acid (0.57 ng/mL, *p* = 0.087) (Supplementary Figure S2B). Amongst participants in the high IL-6 group, the differences were generally larger, with glycocholic acid (adjusted difference = 103.30 ng/mL, *p* = 0.015), taurocholic acid (40.99 ng/mL, *p* = 0.016), and taurochenodeoxycholic acid (33.27 ng/mL, *p* = 0.028) reaching statistical significance. The magnitude of between-period differences decreased from visit 1 to visit 2 and was no longer significant by visit 2 (all *p* > 0.05).

Intervention-by-time interactions were not significant for plasma BA levels (*p* > 0.05 for all) (Figure 4B). Over time, plasma BA concentrations increased in the honey-sweetened yogurt group (glycocholic acid: 20.77 ng/mL, *p* = 0.399; taurocholic acid: 13.20 ng/mL, *p* = 0.156; taurochenodeoxycholic acid: 11.92 ng/mL, *p* = 0.285; tauroursodeoxycholic acid: 0.12 ng/mL, *p* = 0.735), with a smaller increase observed in the sugar-sweetened yogurt group (glycocholic acid: 8.76 ng/mL; taurocholic acid: 2.51 ng/mL; taurochenodeoxycholic acid: 1.19 ng/mL; tauroursodeoxycholic acid: 0.07 ng/mL). A similar pattern was observed in the high IL-6 subgroup (glycocholic acid: adjusted differential change = −55.24 ng/mL, *p* = 0.330; taurocholic acid: −20.93 ng/mL, *p* = 0.354; taurochenodeoxycholic acid: −18.31 ng/mL, *p* = 0.369; tauroursodeoxycholic acid: 0.15 ng/mL, *p* = 0.830), though none reached statistical significance.

### 3.6. Uric Acid

Serum uric acid levels did not differ significantly between the honey and sugar periods at either visit (visit 1: *p* = 0.624; visit 2: *p* = 0.758), and no significant intervention-by-time interaction was observed (*p* = 0.187). This pattern was consistent in both the total sample and the high IL-6 subgroup.

### 3.7. PCA

**Figure 5:**
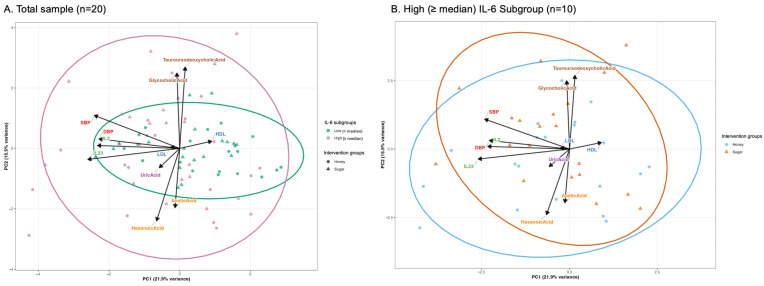
Principal Component Analysis of selected outcomes by interventions and inflammatory burden. PCA plots showing the distribution of fecal metabolite profiles for (**A**) total sample (*n* = 20) and (**B**) participants by IL-6 subgroup assignment (“low” < or “high” ≥ median IL-6 at baseline; *n* = 10). Representative outcomes selected based on correlation matrix. Each point represents an individual sample. Ellipses represent 95% confidence intervals for honey-sweetened yogurt (circle) and sugar-sweetened yogurt (triangle) interventions. Arrows indicate loading vectors for individual metabolites.

PCA was performed to assess multivariate relationships among lipids, SCFAs, BAs, cytokines, and blood pressure measures. The first two principal components explained 37.4% of total variance (PC1: 21.9%, PC2: 15.5%). Variable loadings revealed distinct metabolic patterns: SCFAs loading in the negative PC2 direction, with BA, loading in the positive PC2 direction. SBP, DBP, IL-33 and IL-2 loaded in the negative PC1 direction (Figure 5A). The opposing loadings of SCFAs and BA along PC1 indicate an inverse relationship between fecal SCFA production and circulating BA levels. The co-localization of BA with SBP and IL-2 along negative PC1 suggests coordinated regulation of BA metabolism, BP, and inflammatory signaling. In contrast, the orthogonal loading of DBP and IL-33 along PC2 indicates independent metabolic variation relative to other measured outcomes.

PERMANOVA analysis revealed a significant effect of IL-6 group assignment on overall metabolic profiles (R^2^ = 0.036, F = 2.90, *p* = 0.005), explaining approximately 3.6% of total variance. Despite this statistical significance, there was substantial overlap between the IL-6 groups in multivariate space (Figure 5A). In contrast, intervention effects (honey vs. sugar) on metabolic composition were not statistically significant in either the total sample (R^2^ = 0.013, F = 1.02, *p* = 0.427) or the high IL-6 subgroup (R^2^ = 0.022, F = 0.87, *p* = 0.520), with intervention explaining less than 2.5% of metabolic variance (Figure 5A,B). Homogeneity of dispersion tests validated PERMANOVA assumptions (*p* > 0.10 for all comparisons).

## 4. Discussion

We investigated the impact of 4 weeks of daily intake of a honey-sweetened non-fat probiotic yogurt on pro-inflammatory Th17 cytokines, with a focus on the relationship between cytokine response and microbial-derived SCFA and bile acids. Our intervention did not significantly change our primary outcome measure IL-23. Instead, circulating IL-33 levels were significantly lower after 4 weeks of daily honey-sweetened yogurt intake. In an exploratory aim, significantly greater levels of the immune activator IL-2 were observed after 4 weeks of daily sugar-sweetened yogurt intake. IL-33 is constitutively expressed in endothelial, epithelial and fibroblast-like cells. Considered an alarmin, IL-33 is released upon tissue injury to activate the immune system. IL-33 mediates its response through the binding of ST2 (suppression of tumorigenicity 2) receptors located on a range of immune cell types involved in adaptive and innate immunity [24]. IL-33-induced ST2 expression is dependent on the transcription factor GATA3, maintained in part through IL-2-induction of signal transducer and activator of transcription 5 (STAT5) signaling [24]. Binding of IL-33 to ST2 allows for ST2 interaction with the IL-1 receptor accessory protein (IL-RAcP) to activate several mitogen activated protein (MAP) kinases and nuclear factor (NF)-kB to induce pro-inflammatory cytokine expression [25]. Our study results are in alignment with data reporting reduced IL-33 and MAP kinases expression in mice given a therapeutic level of propolis in a model of atopic dermatitis [26]. Similar to honey, propolis provides the flavanones pinocembrin and pinobanksin and the flavone chrysin, with pinocembrin supplementation reducing NF-kB expression in rodent models [27,28], and chrysin inhibiting the IL-33 induction of NF-kB in vitro [29].

The significant interaction for IL-33 reflects divergent trajectories: increased with sugar-sweetened yogurt but decreased with honey-sweetened yogurt (Figure 3, left panel). This pattern may reflect both active anti-inflammatory effects of honey-derived phenolics (particularly through NF-κB and IL-33 signaling pathway inhibition described above) and potential pro-inflammatory effects of refined sugar. While our design lacks a no-intervention control to definitively distinguish “protection” from “avoidance of harm”, mechanistic evidence for honey’s bioactive compounds to suppress inflammatory pathways [29] suggest an active benefit beyond simply avoiding refined sugar. Clinically, this distinction matters as honey may provide benefits beyond calorie-matched alternatives when used as a sweetener in probiotic-rich foods. In addition, under typical physiological conditions, IL-33 acts as an immune regulator to maintain adipose tissue homeostasis. With increased adiposity this regulation is disrupted, and IL-33 is thought to contribute to a pro-inflammatory phenotype within the adipose [30]. Notably, in our population of predominantly overweight women, we observed increased levels of IL-4, Il-5, IL-13, and IL-10 for those in the high IL-6 subgroup. These cytokines contribute to a Th2 response that promote subcutaneous adipose tissue browning and enhanced thermogenesis [30]. Therefore, in our relatively healthy, but at increased cardiometabolic risk, population, the observed changes in IL-33 may represent a homeostatic adjustment to either intervention, and warrants further study in future dietary interventions.

For human participants, studies examining cytokine levels after daily honey intake have been limited to those with increased oxidative stress [31,32]. Receiving a beverage supplemented with 70 g of unprocessed honey for 8 weeks significantly attenuated IL-1β, IL-6, IL-8 and TNFα levels, while lowering markers of oxidative stress in the seminal fluid of men undergoing 8 weeks of intensive cycling training [32]. In contrast, TNFα levels were significantly increased in smokers consuming 20 g per day of Tualang honey for 12 weeks [31]. However, this study did not report the timing of blood collection relative to an individual’s last smoking period [33]. Our study results provide data towards the often-reported wound healing and anti-inflammatory benefits of honey [34], as well as provide confirmatory data from in vitro and animal models that honey-derived phenolics impact pro-inflammatory cytokine levels through the inhibition of IL-33 cellular signaling pathways [26,29]. However, we enrolled a small sampling of generally healthy individuals with basal cytokine levels often below the limit of detection. These results reduce generalizability to other populations, especially those at higher risk for metabolic disease to include those with obesity and diabetes. The use of self-reported intake for compliance is an additional limitation. Future studies examining the influence of honey intake on cytokine levels in a larger population after peripheral blood mononuclear cell (PBMC) stimulation are warranted, as this will allow, within a healthy population, the detection of both circulating IL-33 levels and expression of associated cytokines.

IL-33 plays a key role in the maintenance of gut homeostasis and protection against pathogenic intestinal infection, with IL-33 dysregulation associated with inflammatory bowel diseases [35]. The immune activation response of IL-33 is inhibited by the SCFA butyrate in animal models and ex vivo in stimulated PBMC [36,37]. The study yogurt for the current study contained probiotic strains along with honey, providing non-digestible oligosaccharides that can contribute to honey’s prebiotic potential in support of SCFA producing species [14,38]. Short-chain fatty acids are predominately produced from intestinal bacteria fermentation of non-digestible polysaccharides, to include fibers and non-resistant starch. In addition to immune regulation, bacterial production of SCFA promotes the integrity, strength and oxidative defense of the intestinal barrier and mucus layer, which limits the systemic entry of endotoxins and other inflammatory mediators that promote chronic disease [39]. Nonetheless, neither yogurt intervention in the current trial significantly impacted fecal SCFA levels, with a lack of confirmatory microbiome data a limitation. Moreover, IL-33 was not associated with fecal SCFA levels and, instead, may be influenced by immune signaling-induced changes by honey-derived flavonoids [27,28].

Our results also suggest that the level of phenolics and oligosaccharides delivered by honey was not sufficient to induce increases in fecal SCFA content. Circulating levels of acetate, butyrate and propionate have been associated with blood pressure regulation, with inverse associations between fecal SCFA and plasma SCFA levels reported [40,41]. While we observed a significant time and treatment interaction for lower blood pressure from baseline after 4 weeks of sugar-sweetened yogurt intake, blood pressure was not associated with fecal SCFA (Figure 5). Instead, the change in blood pressure may represent an overall lowering of blood pressure with yogurt intake independent of sweetener; this was indicated by a significant period effect between the first and last study visits for the whole group (Supplementary Figure S1A). A reasonable observation, given a 3-mmHg reduction in SBP, was reported in a meta-analysis of randomized controlled trials of probiotic fermented dairy intake [42].

Through active and passive transport, approximately 95% of BA are reabsorbed in the distal ileum into the portal vein and enter the enterohepatic circulation [43]. Both the synthesis and enterohepatic circulation of BA are tightly regulated by farnesol X receptors (FXR), a class of nuclear receptors located in the intestine and liver [44]. These receptors play a key role in metabolic health as they regulate both lipid synthesis and BA absorption, as well as glucose homeostasis [43,44]. Bile acids that are not absorbed can be further metabolized by the gut microbiota into secondary BA that are either absorbed or remain in the gut to impact microbial composition and function [43]. Secondary BA can further impact host physiology as agonists for several receptors subtypes [45], with secondary BA such as ursodeoxycholic acid (UDCA) and tauroursodeoxycholic acid (TUDCA) having potential therapeutic use for liver and neurodegenerative disease [46,47]. Part of the therapeutic benefit of these secondary BA is their regulation of the immune response through FXR [48]. Our dataset indicates that the impact of honey intake on IL-33 and IL-2 response was independent of changes in BA production, with neither sweetener impacting BA levels.

Moreover, plasma BA levels were negatively correlated with fecal SCFA. With our current study design, we cannot ascertain whether fecal BA or plasma SCFA behave similarly; however, elevations in both serum SCFA and BA along with increased gut permeability have been observed in diabetics [49]. At baseline, fecal SCFA levels were significantly greater and plasma BA lower in the honey arm, with a trend for lower SCFA and higher BA levels over time with daily honey-sweetened yogurt intake; with the sugar-sweetened yogurt intervention mirroring this response. This observation may represent a change in BA regulation as well as indicate subtle changes in the intestinal epithelium that impacts fecal SCFA absorption. In total, these data demonstrate a need to measure both fecal and circulatory compartments when defining the impact of diet-induced changes on microbial metabolite production.

An important consideration is the substantial added sugar load (~34 g/day) chosen to reflect common yogurt consumption patterns, which exceeds the American Heart Association’s recommendation of ≤25 g/day for women. The observed changes in IL-33 can be weighed against unchanged lipid and glucose profiles. Our small sample size and the 4 week intervention period may have limited detection of a metabolic change. However, a reduction in IL-33 may represent an early response in metabolic regulation [30]. Practically, these findings suggest that when sweeteners are consumed, honey may offer a less inflammatory alternative to refined sugar in probiotic-rich foods, though this should not encourage increased overall added sugar intake above daily recommendations. Future studies in populations with elevated inflammatory burden, using longer durations, functional outcomes, and lower honey levels of honey intake, are needed to determine whether the observed changes in cytokines translate into meaningful clinical outcomes.

## 5. Conclusions

In summary, in a healthy population of postmenopausal women, the daily intake of honey-sweetened yogurt for 4 weeks did not significantly change our primary outcome of IL-23. However, lower circulating levels of IL-33 after 4 weeks of honey-sweetened versus sugar-sweetened yogurt is noteworthy as IL-33 is a pro-inflammatory cytokine that is constitutively expressed in tissues and considered an alarmin, and a key regulator in adipose tissue homeostasis. Moreover, the impact of honey intake on these cytokines was independent of changes in fecal SCFA and plasma BA and may be related to the increased dietary intake of flavonoids known to impact cellular signaling. While our data suggest that, in this generally healthy group of women, yogurt sweetened with honey was less inflammatory than yogurt sweetened with a similar amount of calories from sugar, the intervention provided added sugars (~34 g/day) exceeding recommended limits (≤25 g/day for women). Therefore, these findings should not encourage increased sweetener consumption, but honey may be a preferable alternative. Confirmatory studies, in a larger population and at levels of honey intake that are within dietary recommendations for added sugar intake, are warranted.

## Figures and Tables

**Table 1:** Major bioactive composition (phenolics and sugar) of honey (per 2 tbsp/42 g serving) ^a^.

		Amount (μg per 42 g Serving)
Component	Class	
Phenolics	Phenolic Class	
p-Hydroxybenzoic acid	Hydroxybenzoic acid	98.12 ± 1.38
Caffeic acid	Hydroxycinnamic acid	64.42 ± 0.84
p-Coumaric acid	Hydroxycinnamic acid	147.15 ± 1.26
Pinobanksin-5-methyl ether	Flavanone	140.08 ± 2.64
Pinobanksin	Flavanone	408.86 ± 6.49
Kaempferol	Flavonol	183.29 ± 1.59
Pinocembrin	Flavanone	408.86 ± 6.49
Chrysin	Flavone	115.20 ± 2.13
Galangin	Flavonol	105.84 ± 1.81
5-hydroxymethylfurfural	Furan ^b^	89.14 ± 3.44
Sugar	Carbohydrate Class	Amount (g per 42 g serving)
Fructose	Monosaccharide	15.89 ± 0.65
Glucose	Monosaccharide	13.78 ± 0.55
Sucrose	Disaccharide	0.46 ± 0.02
Trehalose	Disaccharide	0.03 ± 0
Kojibiose	Disaccharide	0.62 ± 0.01
Turanose	Disaccharide	0.60 ± 0.01
Maltose	Disaccharide	1.04 ± 0.01
Isomaltose	Disaccharide	0.21 ± 0.004
Palatinose (isomaltulose)	Disaccharide	0.06 ± 0.004
Nigerose	Disaccharide	0.12 ± 0.004
Melezitose	Oligosaccharides	0.99 ± 0.09
Isomaltotriose	Oligosaccharides	0.01 ± 0.004
Maltotriose	Oligosaccharides	0.13 ± 0.004

^a^ US Grade A Clover Blossoms Honey 2021 with a use-by date of 22 November 2024 [16]. ^b^ Not a phenolic, created from Maillard reaction.

**Table 2:** Participant baseline characteristics by cardiovascular risk and inflammatory burden in the total sample (*n* = 20).

	Total Sample (*n* = 20)	IL-6 Subgroup		
		Low (*n* = 10)	High (*n* = 10)	*p*-Value
Clinicals	Mean ± SD	Mean ± SD	Mean ± SD	
Age (years)	60.05 ± 4.94	60.10 ± 4.72	60.00 ± 5.40	0.965
BMI (kg/m^2^)	28.37 ± 2.72	28.78 ± 3.39	27.96 ± 1.94	0.515
SBP (mmHg)	114.42 ± 8.62	114.03 ± 7.24	114.80 ± 10.20	0.848
DBP (mmHg)	74.73 ± 8.84	76.60 ± 9.80	72.87 ± 7.82	0.359
HR (beats per min)	68.08 ± 7.90	71.07 ± 8.04	65.10 ± 6.89	0.092
TC (mg/dL)	222.27 ± 35.49	225.80 ± 40.11	218.73 ± 31.98	0.668
LDL (mg/dL)	136.20 ± 29.77	137.04 ± 34.50	135.36 ± 26.06	0.903
HDL (mg/dL)	67.08 ± 12.49	69.80 ± 14.42	64.36 ± 10.24	0.344
Non-HDL (mg/dL)	155.19 ± 33.48	156.00 ± 39.14	154.37 ± 28.85	0.917
TC:HDL	3.41 ± 0.72	3.35 ± 0.85	3.47 ± 0.59	0.705
Triglyceride (mg/dL)	86.54 ± 34.18	76.90 ± 40.28	96.18 ± 25.26	0.216
Glucose (mg/dL)	99.71 ± 8.59	97.90 ± 9.47	101.53 ± 7.67	0.359

Data presented as mean ± SD. Subgroups stratified by IL-6 level (“low” < or “high” ≥ median IL-6) at baseline. Statistical comparisons by independent *t*-tests. Abbreviations: SD, standard deviation; BMI, body mass index; SBP, systolic blood pressure; DBP, diastolic blood pressure; HR, heart rate; TC, total cholesterol; LDL, low-density lipoprotein cholesterol; HDL, high-density lipoprotein cholesterol.

**Table 3:** Background dietary intake during intervention periods (excluding yogurt) (*n* = 20).

Nutrient	Honey Period	Sugar Period	*p*-Value
	Mean ± SD	Mean ± SD	
**Macronutrients**			
Energy (kcal/day)	1628.5 ± 427.4	1600.0 ± 357.2	0.634
Protein (g/day)	64.9 ± 22.6	67.7 ± 18.4	0.548
Carbohydrate (g/day)	172.1 ± 56.3	159.9 ± 43.8	0.231
Total Fiber (g/day)	16.1 ± 6.5	14.2 ± 5.9	0.156
Total Sugar (g/day)	61.5 ± 30.6	59.0 ± 23.9	0.627
Added Sugar (g/day)	29.7 ± 23.7	24.9 ± 16.5	0.218
Total Fat (g/day)	71.3 ± 25.0	70.8 ± 21.7	0.891
Saturated Fat (g/day)	24.2 ± 12.0	23.4 ± 9.4	0.697
Monounsaturated Fat (g/day)	17.1 ± 7.5	16.6 ± 6.3	0.686
Polyunsaturated Fat (g/day)	10.2 ± 5.0	9.8 ± 4.8	0.593
**Micronutrients**			
Vitamin C (mg/day)	47.9 ± 30.6	55.4 ± 56.0	0.414
Vitamin D (μg/day)	1.7 ± 2.4	1.1 ± 1.0	0.213
Vitamin E (mg/day)	5.1 ± 3.3	3.9 ± 2.2	0.086
Folate (μg/day)	175.3 ± 80.0	169.2 ± 84.7	0.694
Calcium (mg/day)	564.2 ± 183.7	610.8 ± 350.2	0.432
Iron (mg/day)	9.5 ± 3.4	8.7 ± 3.5	0.234
Magnesium (mg/day)	148.7 ± 64.9	143.6 ± 87.4	0.718
Zinc (mg/day)	4.6 ± 2.1	5.0 ± 2.2	0.312
Potassium (mg/day)	1771.2 ± 647.3	1668.1 ± 549.2	0.395
Omega-3 Fatty Acids (g/day)	1.1 ± 1.0	1.1 ± 0.8	0.985
Omega-6 Fatty Acids (g/day)	8.0 ± 4.7	7.6 ± 4.2	0.554
Sodium (mg/day)	2616.6 ± 866.5	2800.6 ± 838.5	0.254

Data presented as mean ± SD for the total sample (*n* = 20). *p*-values are from paired *t*-tests accounting for the crossover design. No significant differences were observed between intervention periods (all *p* > 0.05).

**Table 4:** Clinical outcomes from descriptive statistics and mixed-effects model results in the total sample (*n* = 20).

	Descriptive Statistics				Mixed-Effects Model Results							
	Honey		Sugar		Intervention β (SE)		Time β (SE)		Interaction β (SE)		Period β (SE)	
Outcomes	Visit 1	Visit 2	Visit 1	Visit 2		*p*		*p*		*p*		*p*
BMI (kg/m^2^)	28.30 ± 2.71	28.27 ± 2.62	28.31 ± 2.84	28.27 ± 2.89	−2.07 (11.75)	0.861	21.69 (12.36)	0.085	−16.54 (16.45)	0.319	−1.28 (0.57)	0.028 *
SBP (mmHg)	110.60 ± 9.63	111.50 ± 11.02	114.51 ± 9.38	110.45 ± 8.14	3.44 (1.59)	0.035 *	2.22 (1.67)	0.189	−4.98 (2.22)	0.029 *	−1.28 (0.57)	0.028 *
DBP (mmHg)	72.55 ± 6.71	71.72 ± 6.42	74.92 ± 9.18	72.62 ± 6.01	2.22 (1.67)	0.190	−0.29 (1.76)	0.870	−1.50 (2.34)	0.524	−0.50 (0.60)	0.409
HR (beats/min)	66.20 ± 7.77	66.13 ± 8.79	68.16 ± 11.10	66.13 ± 7.60	1.95 (1.67)	0.248	0.14 (1.75)	0.935	−1.99 (2.33)	0.397	−0.16 (0.60)	0.788
TC (mg/dL)	217.18 ± 32.67	210.07 ± 32.47	222.44 ± 32.89	210.35 ± 38.11	5.44 (4.75)	0.257	−6.54 (4.99)	0.195	−5.17 (6.64)	0.439	−0.29 (1.70)	0.864
LDL (mg/dL)	131.76 ± 29.12	126.76 ± 26.85	134.21 ± 27.55	129.39 ± 33.79	2.32 (4.25)	0.587	−4.13 (4.47)	0.359	0.08 (5.95)	0.989	−0.73 (1.52)	0.636
HDL (mg/dL)	67.35 ± 12.41	63.86 ± 12.90	67.37 ± 12.35	64.05 ± 12.03	0.41 (1.70)	0.811	−4.45 (1.79)	0.016 *	0.17 (2.38)	0.945	0.96 (0.61)	0.121
Non-HDL (mg/dL)	149.83 ± 32.13	146.22 ± 30.59	155.07 ± 31.73	146.30 ± 36.89	5.10 (4.15)	0.224	−2.09 (4.35)	0.634	−5.37 (5.79)	0.358	−1.22 (1.49)	0.417
TC:HDL	3.33 ± 0.79	3.43 ± 0.84	3.41 ± 0.72	3.37 ± 0.77	0.07 (0.09)	0.468	0.16 (0.10)	0.104	−0.14 (0.13)	0.288	−0.06 (0.03)	0.095
Triglyceride (mg/dL)	91.50 ± 38.85	86.06 ± 20.97	92.93 ± 39.48	87.90 ± 34.17	2.48 (8.71)	0.776	−7.83 (9.16)	0.396	0.36 (12.18)	0.977	2.46 (3.11)	0.433
Glucose (mg/dL)	98.60 ± 8.81	100.98 ± 8.34	100.36 ± 11.59	99.26 ± 8.20	1.96 (2.13)	0.362	2.08 (2.24)	0.357	−3.52 (2.98)	0.242	0.35 (0.76)	0.644

Data presented as mean ± standard deviation for each intervention at visit 1 and visit 2, followed by mixed-effects model results presented with regression coefficients β and standard error, with honey period as a reference. Mixed models included intervention, time, sequence, period, and baseline weight as fixed effects, with subject as a random effect. Intervention β represents the effect of sugar-sweetened yogurt compared to honey-sweetened yogurt. Time β represents the effect of visit 2 compared to visit 1 (baseline). Interaction β represents the intervention-by-time interaction effect. Abbreviations: SE, standard error; BMI, body mass index; SBP, systolic blood pressure; DBP, diastolic blood pressure; HR, heart rate; TC, total cholesterol; LDL, low-density lipoprotein cholesterol; HDL, high-density lipoprotein cholesterol. * *p* < 0.05.

## Data Availability

The raw data supporting the conclusions of this article will be made available by the authors on request.

## Supplementary Materials

The following supporting information can be downloaded at: https://www.mdpi.com/article/10.3390/nu18030522/s1, Table S1: Participant baseline cytokine characteristics stratified by IL-6 level in the total sample (*n* = 20); Figure S1: Period effect on systolic blood pressure and hexanoic acid in crossover trial; Figure S2: Intervention effects (honey vs. sugar) at baseline on fecal short-chain fatty acids and bile acids.
