# Supplementary material for: The Influence of Daily Honey-Sweetened Yogurt Intake on Outcomes of Low-Grade Inflammation and Microbial Metabolites in Postmenopausal Women
Source: Nutrients. 2026 Feb 4;18(3):522. doi: 10.3390/nu18030522 (PMC12899863; doi:10.3390/nu18030522)
Supplement: Supplementary file 1 [file nutrients-18-00522-s001.zip › nutrients-4072583-supplementary.pdf]

Supplementary Table S1. Participant baseline cytokine characteristics stratified by IL-6 level in the total sample (n=20).

	**Total sample (n=20)**	**IL-6 Subgroup**		
		**Low (n=10)**	**High (n=10)**	**p-value**
Cytokines (Categorical)	*n (%)*	*n (%)*	*n (%)*	
**IL-17F detected**	5 (25.00)	2 (20.00)	3 (30.00)	1
**IL-17E/IL-25 detected**	3 (15.00)	0 (0.00)	3 (30.00)	0.211
**IL-31 detected**	6 (30.00)	0 (0.00)	6 (60.00)	**0.011***
Cytokines (Continuous)	*Mean ± SD*	*Mean ± SD*	*Mean ± SD*	
**GM-CSF (pg/mL)**	0.04 ± 0.05	0.02 ± 0.04	0.06 ± 0.05	0.083
**IFN-γ (pg/mL)**	2.94 ± 3.88	2.50 ± 3.82	3.38 ± 4.09	0.625
**IL-10 (pg/mL)**	4.55 ± 3.83	2.10 ± 2.20	**7.00 ± 3.59**	**0.002****
**CCL20/MIP-3α (pg/mL)**	24.03 ± 19.53	18.49 ± 21.68	29.57 ± 16.35	0.213
**IL-12p70 (pg/mL)**	9.75 ± 14.84	10.12 ± 20.20	9.38 ± 7.54	0.915
**IL-13 (pg/mL)**	12.56 ± 14.99	2.61 ± 4.22	**22.52 ± 15.37**	**<0.001*****
**IL-15 (pg/mL)**	4.90 ± 3.60	3.74 ± 2.53	6.06 ± 4.24	0.155
**IL-17A (pg/mL)**	2.02 ± 2.49	1.23 ± 2.54	2.81 ± 2.29	0.160
**IL-22 (pg/mL)**	0.21 ± 0.36	0.07 ± 0.15	0.36 ± 0.46	0.071
**IL-9 (pg/mL)**	8.86 ± 12.53	7.59 ± 16.07	10.13 ± 8.33	0.662
**IL-1β (pg/mL)**	3.43 ± 5.49	3.07 ± 6.94	3.79 ± 3.89	0.776
**IL-33 (pg/mL)**	8.37 ± 14.00	2.65 ± 6.88	14.08 ± 17.15	0.066
**IL-2 (pg/mL)**	2.43 ± 2.26	1.76 ± 2.24	3.11 ± 2.19	0.189
**IL-21 (pg/mL)**	11.88 ± 29.45	15.27 ± 41.86	8.49 ± 7.28	0.620
**IL-4 (pg/mL)**	0.14 ± 0.18	0.01 ± 0.02	**0.28 ± 0.16**	**<0.001*****
**IL-23 (pg/mL)**	0.67 ± 0.90	0.26 ± 0.47	**1.08 ± 1.06**	**0.037***
**IL-5 (pg/mL)**	11.16 ± 12.04	2.04 ± 2.83	**20.28 ± 10.65**	**<0.001*****
**IL-6 (pg/mL)**	15.20 ± 17.64	1.33 ± 1.81	**29.07 ± 15.05**	**<0.001*****
**IL-27 (pg/mL)**	0.30 ± 0.14	0.29 ± 0.19	0.31 ± 0.09	0.687
**TNF-α (pg/mL)**	15.63 ± 5.15	15.17 ± 6.30	16.08 ± 3.97	0.705
**TNF-β (pg/mL)**	0.21 ± 0.25	0.02 ± 0.03	**0.41 ± 0.22**	**<0.001*****
**IL-28A (pg/mL)**	1.04 ± 1.15	0.12 ± 0.17	**1.95 ± 0.94**	**<0.001*****

Data presented as mean ± SD. Groups stratified by IL-6 level (low < or high ≥ median IL-6) at baseline. Statistical comparisons by independent t-tests. Abbreviations: SE, standard error. *p<0.05, **p<0.01, ***p<0.001.

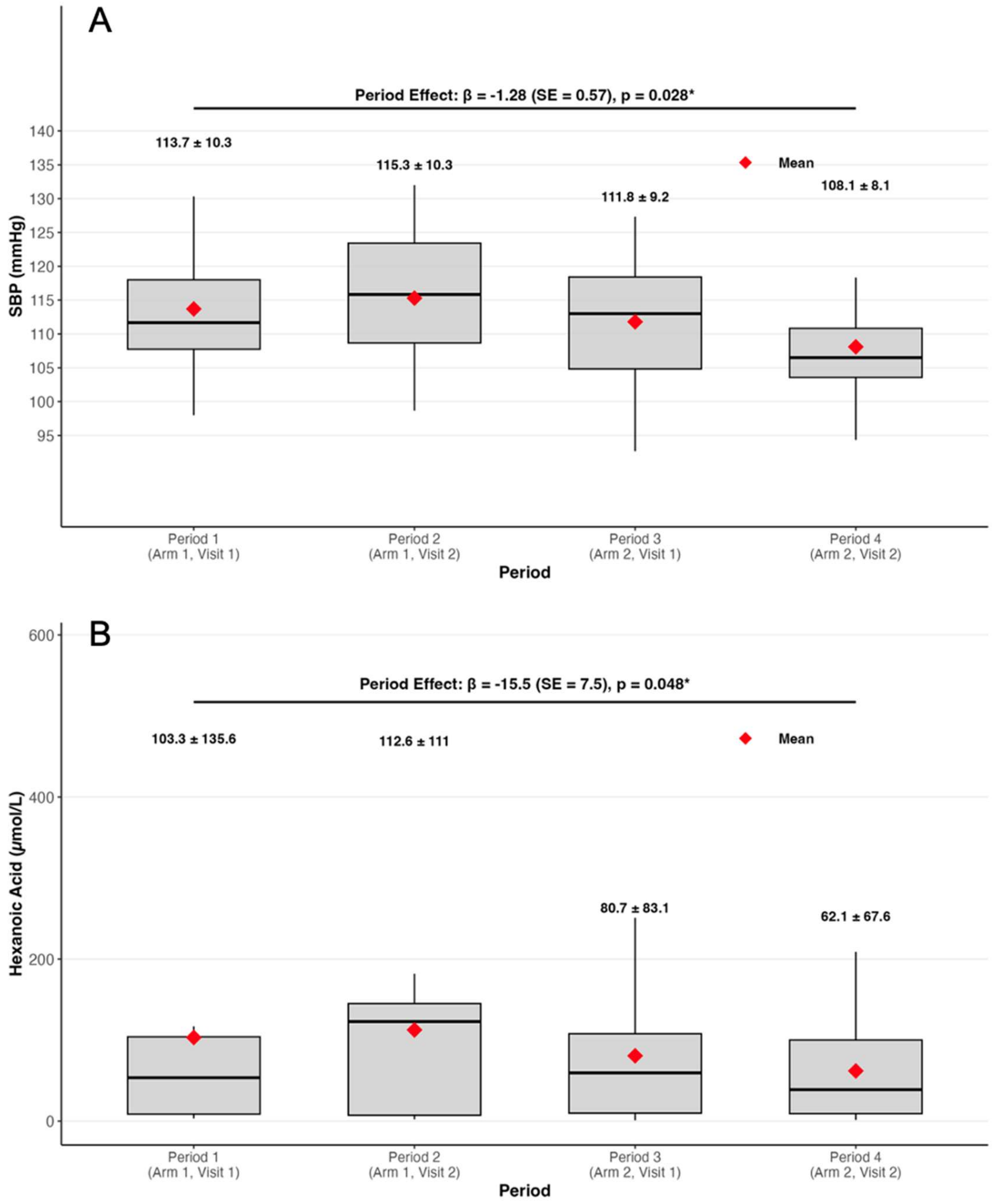

Supplementary Figure S1. Period effect on systolic blood pressure and hexanoic acid in crossover trial. Systolic blood pressure (SBP) measurements (A) and hexanoic acid concentrations (B) across the four study periods in a crossover design (n=20). Boxes represent the interquartile range (IQR, 25th-75th percentile), horizontal lines indicate medians, and red diamonds indicate means. Values above boxes show mean ± SD. Results from linear mixed-effects models adjusted for time, sequence, period, and weight, with honey period as reference. * $p<0.05$.

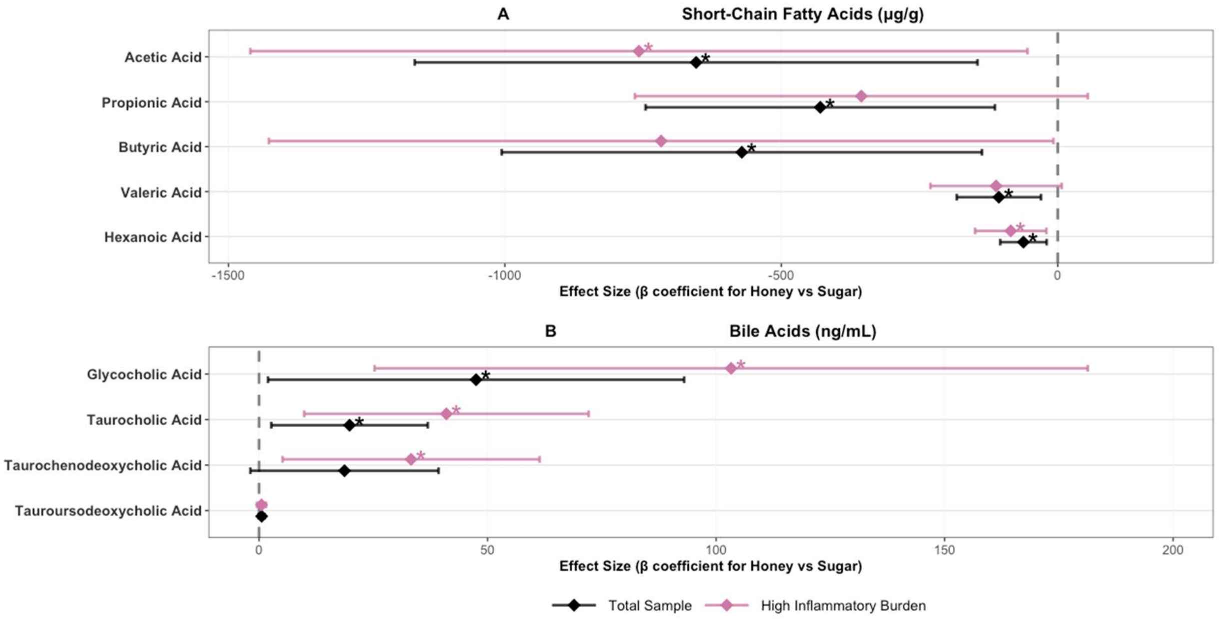

Supplementary Figure S2. Intervention effects (honey vs. sugar) at baseline on fecal short-chain fatty acids and bile acids. Forest plot showing β coefficients with 95% confidence intervals comparing honey- vs. sugar-sweetened yogurt effects on (A) fecal short-chain fatty acids (μg/g) and (B) bile acids (ng/mL). Black: total sample (n=20); Pink: high IL-6 subgroup (n=10). Dashed line indicates no effect (β=0). Results from linear mixed-effects models adjusted for time, sequence, period, and weight, with honey period as reference. Positive β indicates higher concentrations in sugar vs. honey group. *$p < 0.05$.
